# Shaping social behavior in an enriched environment

**DOI:** 10.3389/fnbeh.2022.999325

**Published:** 2022-10-13

**Authors:** Liliana Amorim, Sandro Dá Mesquita, Luís Jacinto, Magda J. Castelhano-Carlos, Nadine Correia Santos, Hugo Leite-Almeida, Nuno Sousa

**Affiliations:** ^1^Life and Health Sciences Research Institute (ICVS), School of Medicine, University of Minho, Braga, Portugal; ^2^ICVS/3B’s – PT Government Associate Laboratory, Guimarães, Portugal

**Keywords:** social behavior, rats, enriched environment, impulsivity, competition

## Abstract

Access to vital needs shapes social orders. In rats, social systems tend to maintain a certain stability, but alterations in the physical environment can change inter-individual relations, which consequently can alter social orders. Principles governing social systems are, however, difficult to study and most analyses have been restricted to dyads of animals over short periods of time, hardly capturing the complexity and temporal dynamics of social interactions. Herein, we studied social interactions in a colony of six rats living in a customized enriched environment (PhenoWorld, PhW), under variable conditions of access/availability to limited resources. Reductions in food accessibility and availability resulted in a marked heterogeneity in sniffing, chasing and fighting/struggling behaviors, and, in the latter condition, an overall increase of these displays. The introduction of the possibility of interaction with a female rat also increased the amount of sniffing and fighting/struggling in a homogeneous manner. Results also showed that individual food retrieval success had no association with fighting/struggling when food pellets are delivered to the animals. However, there was a statistically significant correlation between fighting/struggling and impulsivity as measured by the amount of premature responses in the Variable-to-Signal-Test outside of the PhW providing external validation to our measures. To sum up, through continuous monitoring of a group of rats in the PhW, we demonstrated how variations in access to reinforcers modulate social behavior.

## Introduction

Rodents typically live in groups in natural conditions and have to share food and water resources that are scattered through their domain. Rats, in particular, can express complex behavior and tend to form complex social systems modulating their behavior in function of the social environment (Grant and Chance, 1958; Blanchard et al., 1988; Malatynska and Knapp, 2005; Himmler et al., 2013). Social interplay consists of positive and negative interactions and both contribute to the establishment and maintenance of social structure within the group of animals (Malatynska and Knapp, 2005). In a laboratory setting, however, single and double animal housing is typically favored against group housing. Thus, analysis of social interactions, either manual or automated, is usually restricted to pairs of animals either in their home cage or in novel interactive arenas designed to assess one-way interactions, typically in very short periods of time (Silverman et al., 2010; Castelhano-Carlos et al., 2014, 2017; Beery et al., 2020). The main pitfall of these approaches is that baseline complex social interplay – and consequent group structure – and time-dependent modulations with functional consequence are lost, leading to limited conclusions. Analysis of social interactions in small groups of animals living in complex environments, that may better reflect natural conditions is rarely performed in laboratory conditions. Proper analysis of normal and deviant social interplay is, however, critical to better understand several neuropsychiatric disorders and respective neurobiological underpins (Kozorovitskiy and Gould, 2004; Wesson, 2013; Opendak et al., 2016).

Here, we longitudinally assessed how social interplay and group structure profiles were affected by variable degrees of food accessibility in groups of rats living permanently in a customized and validated ethological enriched environment (Castelhano-Carlos et al., 2014). Additionally, we also assessed how changes in social interplay benefited the success of food access in a competitive context. Finally, we attempted to generalize our observations using a stimulus of a different nature but that could possibly generate competition. Specifically, we measured the impact of a conditioned access to a female rat in social behavior and group structure.

## Materials and methods

### Animals and behavioral assessment in the PhenoWorld

The PhenoWorld (PhW; TSE Systems GmbH, Bad Homburg, Germany) is a validated multimodal caging and testing system for rats that allows uninterrupted collection of individual data, reducing human interference (Castelhano-Carlos et al., 2014, 2017). It consists of a square central compartment (1 m^2^ × 50 cm height) connected to several additional modules. During our experiment, these included a compartment with four running wheels and two independent dinking/feeding compartments.

Six Wistar Han male rats (Charles River Laboratories; France), 3 months-old at the beginning of the experiment, were housed in the PhW under standard laboratory conditions: artificial 12 h light/dark cycle (inverted light cycle - lights off 10 a.m.), 21 ± 1°C ambient temperature and 50–60% relative humidity. Corncob was used as bedding material (Scobis Due; Mucedola SRL, Settimo Milanese, Milan, Italy) and sterile cardboard tubes as housing refinement. Rats were given a standard diet (4RF21; Mucedola S.R.L., Italy) and water *ad libitum*. Health monitoring was performed in compliance with the Federation of European Laboratory Animal Science Associations (FELASA) guidelines (Nicklas et al., 2002). All animals were implanted with a subcutaneous RFID transponder (Planet ID GmbH, FDX-B; Germany) for individual identification and monitorization as described before (Castelhano-Carlos et al., 2014). Briefly, the skin on the middle dorsal area of the animal, site of placement for the RFID transponder, was previously anesthetized by subcutaneous injection of 250–300 μl (100 μl per 100 g body weight) of 0.5% Lidocaine (from 2% Lidocaine solution, B. Braun Medical Lda, Queluz de Baixo, Portugal), using a 25-G (25 mm long) needle. A RFID transponder, 12 mm long × 2.12 mm diameter, 0.09 g weight, covered with Bio Glass 8625 and inserted in a 2.6 mm × 32 mm needle, was then subcutaneously injected with the help of a transponder injector (injector and Yellow label transponder from Planet ID; ISO FDX-B Standard/manufacturer code 972). Access to the drinking/feeding and female compartments was controlled by automatic animal gates (AG) equipped with an RFID antenna/reader for identification and register. Only one animal at a time was permitted in these areas except when indicated (single-, double-, and no-delivery paradigms; indicated by double arrow in the images). Rats were habituated to the steady state conditions of PhW (i.e., individual access through transponder-activated automated gates), for 4 weeks. After this period, animals were daily monitored for five consecutive days (for 24 h) with the help of a surveillance video camera installed above the home cage of the PhW. To avoid the influence of habituation to the novel paradigm, the activity and behaviors were analyzed only in the 5th day of recordings. For visual discrimination, rats were marked in the tail (stripes) and in the back (spots). General activity was quantified in blocks of 5 mins over 24 h by a 6 items scale: 5 – all rats moving constantly; 4 – at least three rats moving constantly; 3 – more than three rats moving regularly; 2 – rats grooming and/or less than three rats moving regularly; 1 – rats in central cage but not moving (resting/sleeping); 0 – no rats in the central cage. The different types of social behaviors taking place in the home cage were analyzed; these included: allogrooming, sniffing, interaction in gates and standing near gates, chasing, fighting/struggling, on-top and on-bottom. The frequency of each behavior was assessed in blocks of 5 mins, during the dark phase (10–22 h).

### Experiment 1: Paradigm 2- to 1-gate

Rats were filmed for five consecutive days under normal PhW’s feeding/drinking conditions, which consist on individual access via transponder-activated automated gates, to the two cages containing food and water *ad libitum*. To analyze the influence of decreased access to food and water, the entrance to one of the feeding/drinking cages was prevented. General activity and social behavioral were analyzed.

### Experiment 2: Scheduled delivery (single and multiple) paradigms

First, food was removed from the feeding/drinking cages and the automatic gates were open to allow free-access to the drinking bottles. Rats were given 12 food pellets (≈4,8 g/pellet), i.e., 2 per rat, at the mid of the dark period (16 h), for five consecutive days via an automatic motorized feeder (single deliver condition, s.d.). Then, the feeder was programmed to deliver three food pellets, i.e., 1 per two rats, every to 2 h during the dark period, also for a 5 days period (multiple deliver, m.d.). Food delivery in this paradigm was paired with a sonorous tone. A final session was performed in extinction, in which no food was delivered but the tone was maintained (no deliver, n.d.). In all cases behavior was monitored and analyzed.

### Experiment 3: Female assay

For this condition, one of the drinking cages was replaced by a cage containing an enclosed female rat, whose access was conditioned to a single rat at a time by a transponder-activated automated gate, similarly to the previous conditions. Animals were feed once a day 12 food pellets (2 pellets per rat, at the mid of the dark period – 16 h). Behavior was monitored and analyzed.

### Experiment 4: Variable delay-to-signal test

The variable delay-to-signal (VDS) was performed as described by the group (Leite-Almeida et al., 2013; Soares et al., 2018) aiming to obtain behavioral correlates o impulsive behavior. Briefly, the VDS was performed outside the PhW, in 5-hole chambers (TSE Systems, Germany). The protocol consisted of 10 training sessions (twice daily, 5 h apart), 100 trials (or 30 mins) each. Trials started with the lightning of the house light for 3 s (delay period) followed by the light stimulus in the response aperture for 1 min (response period). Nose pokes in the response period were reinforced with the delivery of a sugar pellet (timed nose pokes) while responses in the delay period (premature responses) were punished with a timeout period (5 s) in complete darkness. The VDS itself consisted in a single 120 trials session starting with an initial block of 25 trials at 3 s delay (3 si) followed by 70 trials at 6 (6 s) or 12 s and again a final block with 25 trials with 3 s delay (3 sf). Premature responses and multiple nose pokes were not punished and multiple nose pokes were allowed. The amount of premature responses per minute of delay was then calculated.

## Results and discussion

### Accessibility to food influences PhenoWorld hierarchy

**FIGURE 1:**
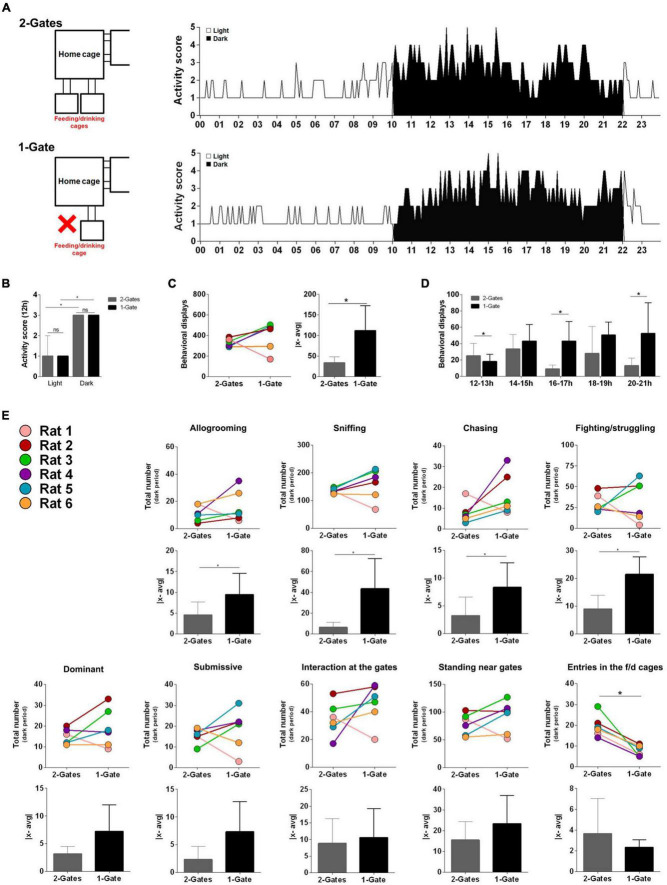
Activity in the home cage: 2- and 1-gate conditions. **(A)** Representative graphs of the activity of the six rats, in the home cage, in the 2- and 1-Gate paradigm. **(B)** Results of activity scores in the central cage were similar in the 2- and 1-Gate experiments; scores were significantly higher in the dark period. Results are presented as median and IQR; *n* = 144 scores/group, Wilcoxon test. **(C,D)** Access reduction to food significantly increased the number of behavioral displays (particularly evident in the later phases of the dark period), as well as the dispersion within the group. **(E)** 2- to 1-Gate transition significantly increased within group variability in allogrooming, sniffing, chasing and fighting/struggling. Results are presented as median and IQR; *n* = 6/group, *p < 0.05, Wilcoxon test.

Social animals, when housed together, establish hierarchies (Wang et al., 2014). While generally accepted that such social relations are plastic, the principles governing hierarchization are poorly understood. Considering the close relationship between food-seeking and foraging behaviors and the ability of different organisms to learn, adapt, reproduce and survive, e.g., bumble-bees (Raine and Chittka, 2008), rats (Kim et al., 2015), and seals (Jeanniard-du-Dot et al., 2017) – we explored whether different competitive conditions of food accessibility/availability would impact on social hierarchization. To determine this, we housed a group of rats in an enriched environment since a young age and manipulated, in a controlled manner, animals’ access to food. As expected, activity scores were significantly higher in the dark period when compared to the light period (*Z*_2–Gates_Light–Dark_ = −9.029, *p* < 0.001; Figures 1A,B), and the total amount of behavioral displays was relatively homogeneous (Figure 1C). When the condition was changed from 2- to 1-Gate, the activity scores maintained light/dark relation (*Z*_1–Gate_ = −9.436, *p* < 0.001; Figure 1B), the activity scores during the dark phase were higher in the latter condition (*Z*_12–13 h_ = −1.997, *p* = 0.046; *Z*_14–15 h_ = −0.314, n.s.; *Z*_16–17 h_ = −1.992, *p* = 0.046; *Z*_18–19 h_ = −1.214, n.s.; *Z*_20–21 h_ = −2.201, *p* = 0.028; Figure 1D) and the behavioral heterogeneity increased [*Z*_|x–avg|_ = −2.221, *p* = 0.028; Figure 1C]. Regarding specific behavioral displays, no overall differences were observed in the group median, though a statistically significant increase in dispersion was observed – allogrooming [*Z*_|x–avg|_ = −1.992, *p* = 0.046], sniffing [*Z*_|x–avg|_ = −2.221, *p* = 0.028], chasing [*Z*_|x–avg|_ = −2.014, *p* = 0.044] and fighting/struggling [*Z*_|x–avg|_ = −1.992, *p* = 0.046; Figure 1E]. Decreased food availability therefore resulted in a dynamic alteration in the interindividual relations though it is not entirely clear if the observed heterogeneity increase parallels an hierarchization of the group. Indeed, ranks vary in sniffing, chasing and fighting/struggling with no evident leader standing out. In line with our observations, a previous study reported an increase in the number of aggressive actions in an otherwise stable group of rodents living in a visible burrow system (Blanchard et al., 2001) after the exchange of the dominant rats (Opendak et al., 2016). It is relevant to note that in our paradigm, and in contrast with other reports on this topic (Blanchard et al., 1988; Kozorovitskiy and Gould, 2004; Opendak et al., 2016), the initial population was formed by young adult male siblings in which social hierarchies were not particularly evident, i.e., the group was fairly homogeneous at the baseline conditions. One interesting aspect of the 2- to 1-Gate variation in our paradigm is that it clearly fostered these hierarchies and provided an individual and group analysis of its setting. Importantly, such analysis revealed that it affected not only behaviors that are classically associated with dominance/submission – lateral attacks, on-top and chasing (Takahashi and Blanchard, 1982) – but it also affected sniffing. In an elegant work using radio-telemetric recordings of nasal respiration, Wesson reported that this behavior was used not only to receive but also to convey socially relevant information (Wesson, 2013). Specifically, subordinate animals decrease sniffing when inspected by dominant animals and a failure to do so leads to a decreased latency to agonistic attacks from the latter (Wesson, 2013). Indeed, sniffing behavior was very homogenous in the PhW inmates in the basal condition, but changed markedly when access to food was restricted to 1-gate. Furthermore, animals ranking higher in the classical measures of hierarchy, e.g., fighting/struggling, presented also higher sniffing behavior.

### Distinct patterns of food delivery modulate social interactions

**FIGURE 2:**
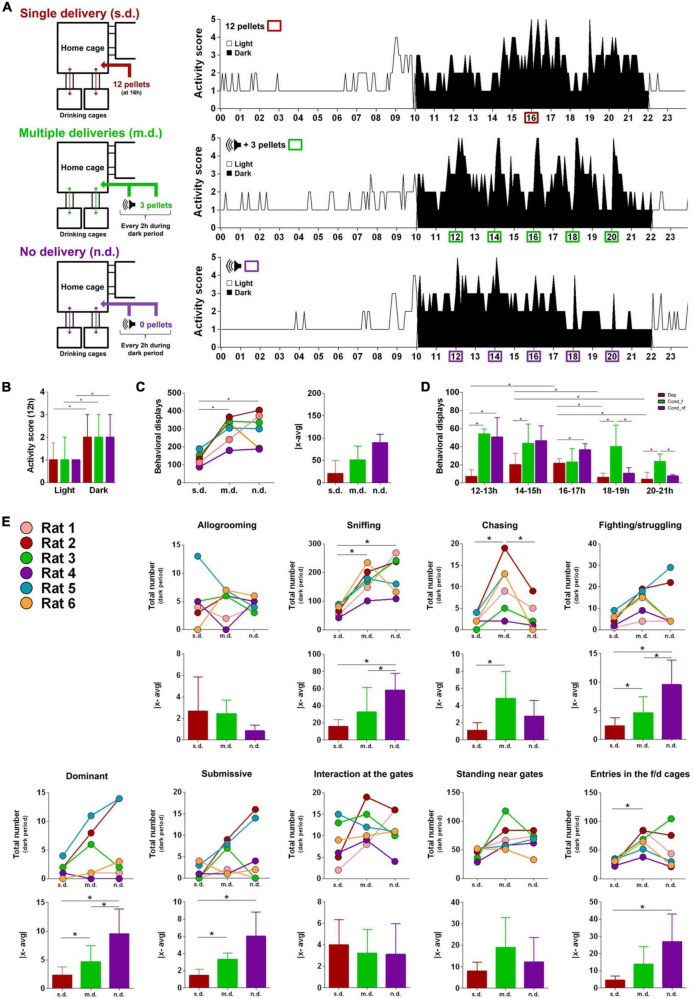
Activity in the home cage: single and multiple deliveries conditions. **(A)** Representative graphs of the activity score of the six rats in the home cage, in the single delivery (s.d.; 2 pellets/rat at 16 h), multiple deliveries (m.d.; 1 pellet/2 rats every 2 h paired with a sonorous tone) and no delivery paradigms (n.d.; sonorous tone every 2 h); events are represent with a colored square; brown, green, and purple, respectively. **(B)** m.d. condition increased activity scores in the dark period but not in the light period. **(C,D)** m.d. deliveries condition significantly increased the number of behavioral displays without any evident effect on the dispersion of the groups; in the n.d. activity/displays decreased as the tone association extinguish. **(E)** Sniffing and chasing significantly increased (number and dispersion) in the m.d. condition. Results are presented as median and IQR; *n* = 6/group, *p < 0.05, Wilcoxon test.

In a novel set of conditions, food was provided in PhW’s central living cage to all animals simultaneously (single deliver, s.d.; Figure 2A, top left). First, we removed food pellets from their receptacles in the feeding/drinking cages (thereon called drinking cages) and we maintained the automatic gates open, allowing multiple-animal unrestrained access to the drinking bottles (bidirectional arrows, Figure 2A, top left). In this condition, an automatic feeder delivered a single daily amount of 12 pellets (i.e., 2 pellets per rat) in a specific location of the central cage. This was later followed by two additional conditions, one on which a reduced amount of food (one pellet per two rats) was delivered every 2 h paired with an audible tone (multiple delivers, m.d.; Figure 2A, middle left) and then a condition in which no food was delivered with the tone (no deliver, n.d.; Figure 2A, bottom left) eventually leading to extinction of the acquired response. Again, in all conditions, activity scores were significantly higher during the dark phase (*Z*_s.d._ = −8.310, *p* < 0.001; *Z*_m.d._ = −8.400, *p* < 0.001; *Z*_n.d._ = −7.208, *p* < 0.001; Figure 2B). The introduction of the s.d. condition resulted in a decrease in the number of behavioral displays compared to the 2-Gate condition (Median s.d. = 151 vs Median 2-Gate = 350; Figure 2C, see also for comparison Figure 1C). Also, in this s.d. condition, the hierarchic distinction between PhW housing mates became less clear; in fact, no distinction could be attained based on sniffing, chasing and fighting/struggling displays (Figure 1E). One possible explanation for the overall reduction in activity may relate with animals’ energy expenditure. However, several studies showed that caloric restriction is paradoxically associated with an increase in activity – see for instance (Chen et al., 2005; Ruegsegger et al., 2016; Acosta-Rodriguez et al., 2017). In the m.d. and n.d. assays, comparatively to the s.d., a generalized increase of activity scores and behavioral displays was observed throughout the entire dark period (Figure 2D). Again, sniffing and chasing behavioral displays were the most affected, presenting a significant increase in the number (Z_Sniffing_:s.d.-m.d. = −2.201, *p* = 0.028; Z_Sniffing_:s.d.-n.d. = −2.201, *p* = 0.028; Z_Chasing_: s.d.-m.d. = −2.023, *p* = 0.043; Z_Chasing_: m.d.-n.d. = −2.207, *p* = 0.027) and in the dispersion of the values (Z_Sniffing_: s.d.-m.d. = −2.201, *p* = 0.028; Z_Sniffing_: s.d.-n.d. = −2.201, *p* = 0.028; Z_Chasing_: s.d.-m.d. = −2.201, *p* = 0.028; Figure 2E). This set of experiences reveals that higher competitive conditions are associated with an increase in social displays, namely to those related with the establishment of social hierarchies. Experimental settings bearing some resemblances with ours have been used in several other studies (Gentsch et al., 1988a,b, 1990; Joly and Sanger, 1991, 1992; Woodall et al., 1996; Malatynska et al., 2002; Hoshaw et al., 2006; Millard and Gentsch, 2006; Lofgren et al., 2013), and despite their limitations, in those paradigms dominant/subordinate relations could also be readily distinguished. Interestingly in the n.d. condition, in which the tone was dissociated from food delivery, we observed a decrease across the dark period of both activity scores (Figure 2A) and behavioral displays (Figure 2D) indicating the extinction of the behavior and therefore the specificity of the alterations induced by the conditioned condition. Curiously, sniffing maintained a high value probably as a result of direct inspections between the animals (Figure 2E).

### Access to interaction with a female increase sniffing and fighting/struggling

**FIGURE 3:**
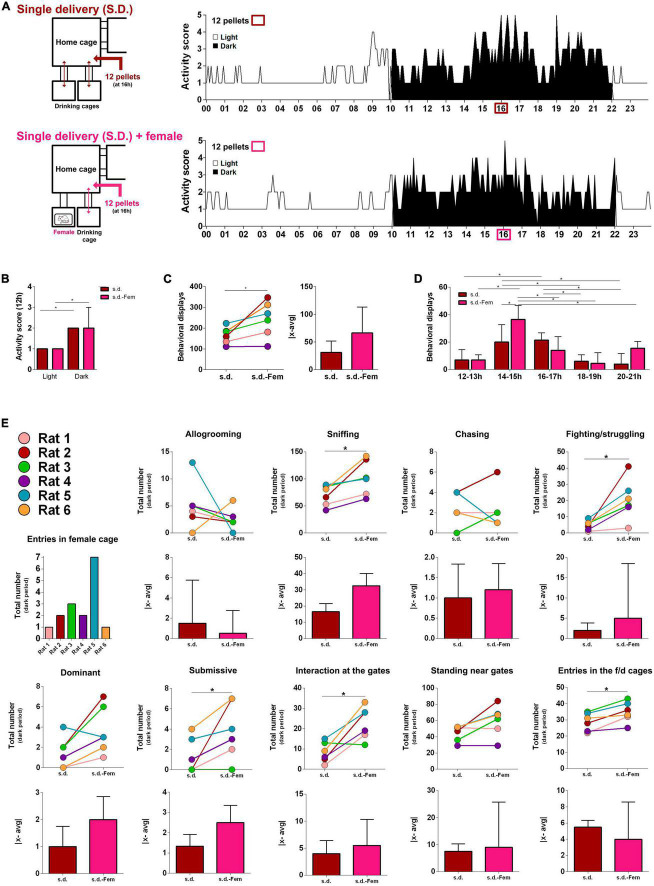
Presence of a female rat cage induced an increase in the number of behaviors. **(A)** Representative graphs of the activity score of the six rats, in the home cage, in the deprived (single daily deliver of 2 pellets/rat at 16 h) and deprived in the presence of a female. **(B)** The presence of a female had no effect on the activity scores in the dark and light periods but **(C,D)** increased the number of behavioral displays; **(E)** specifically sniffing, fighting/struggling and submissive, interaction at the gates and entries in feeding/drinking cage. Results are presented as median and IQR; *n* = 6/group, *p < 0.05, Wilcoxon test.

While in the s.d. condition (a single food delivery at 16 h), we modified one of the drinking cages so that it contained a cage with a female rat throughout the 5 days of the experiment (Figures 3A–D). Access to interaction with the female was conditioned to a single rat at a time by the transponder-activated automated gate (Figure 3A). Indeed, even maintaining the s.d. setting, the introduction of the female cage induced a significant increase in the total number of behaviors/interactions of each rat (*Z* = −2.201, *p* = 0.028; Figure 3C). This effect was mostly due to the significant increase in the number of sniffing (*Z* = −2.201, *p* = 0.028), fighting/struggling (*Z* = −2.201, *p* = 0.028) and on-bottom (*Z* = −2.032, *p* = 0.042) behaviors as well as interactions at the gates (*Z* = −1.997, *p* = 0.046), i.e., behaviors directly associated with the access to the female cage (Figure 3E). Interestingly, no major alterations were found in the dispersion measures (Z_*total*|x–avg|_ = −1.572, *p* = 0.116; Figure 3C) suggesting that all individuals changed similarly. Previous reports have also shown increased inter-male aggressiveness in the presence of a female, even without direct male-female contact (Taylor, 1980; Taylor et al., 1987; Koyama and Kamimura, 2000). Of note, herein we show that the presence of a female rat alone, without any change in food availability, was enough to increase the number of actions, namely of sniffing, fighting/struggling, on-bottom and interactions at gates (Figure 3E). This indicates that the shift observed in the s.d condition (Figure 2) was of social nature rather than a metabolic effect. These results reveal that it is possible to increase the male rats’ overall activity, even in a scenario of caloric restriction, by simply introducing a new stimulus. They also reveal that competition can be triggered by different types of incentives. Finally, this set of data reveals that animals when disputing for desired objectives readjust social interactions, becoming more heterogeneous in several behavioral readouts and eventually more hierarchized.

### Aggressiveness and impulsivity behavior are correlated

Impulsivity and aggressive behaviors have often been found to associate (Cervantes and Delville, 2007). Therefore, in order to provide an external validation to our PhW assessment we tested for impulsivity in the Variable Delay-to-Signal test – VDS (Leite-Almeida et al., 2013; Soares et al., 2018); see also for review (Esteves et al., 2021). In this paradigm animals learn to wait for a light signal for 3 s in order to obtain a reinforcer after a timed nosepoke. After 10 sessions animals are exposed to trials with long (and variable) delays of 6 and 12 s; delay intolerance manifests in an increased response rate correct for the amount of available delay.

**FIGURE 4:**
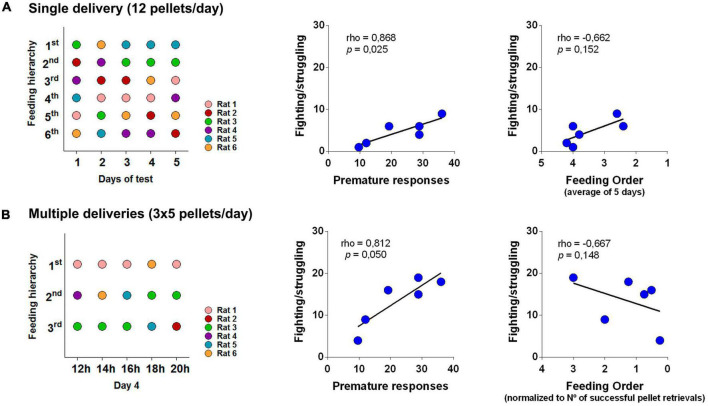
Fighting/struggling correlates with external measures of impulsive behavior. **(A,B)** Feeding hierarchy was determined as the order by which rats reached and grabbed their first food pellet upon delivery by the automatic motorized feeder in each day of the test (s.d.) or in each deliver of the 4th day (m.d.). **(A)** Feeding ranks in s.d. were calculated as the average of the order of pellet retrieval in the 5 days of the experiment; in m.d. **(B)** feeding ranks were calculated as the average of the order of pellet retrieval/No of successful pellet retrievals ratio. In both conditions, the counting of behavioral displays of fighting/struggling positively correlated with impulsive behavior assessed by the variable delay-to-signal (VDS) test. No relation was found between this behavioral display and feeding hierarchy. The Spearman’s correlation test was used to perform the analysis and *p*-value (two-tailed). Correlation was considered significant for *p* < 0.05, *n* = 6.

Indeed, statistically significant correlations were found between the number of fighting/struggling behaviors and VDS prematurity in both s.d. and m.d/n.d. conditions (Figures 4A,B, respectively) providing an important external validation. Feeding order scores did not, however, correlate with the amount of fighting/struggling displayed by the animal in the s.d. and m.d. Such was particularly evident in the latter condition, indicating that when access to valuable item was strongly limited (1 pellet per two animal at each release) the hierarchical relations were no longer observed, i.e., individually, animals actively seek to adapt to a highly competitive condition and success is not determined by social hierarchy.

## Conclusion

Social gradings are largely dictated by access limitations to basic needs. While our work does not aim to model the complexity and idiosyncrasies of human societies, it is clear that the transformations observed in the PhW both at group and individual levels have an obvious construct value. We showed that social interactions in the PhW are highly plastic being contingent with transitory alterations in the environmental conditions. Despite the small number of individuals in the setting (dictated by the characteristics of the apparatus) the behavioral alterations were consistent across multiple challenges namely food access and availability to food, but also access to social stimulus (in this case, interaction with a female). Individually, all modulated the amount and heterogeneity of behavioral interaction between rats, reflecting on the process of social hierarchy. Overall, the PhW may be a suitable environment for the study of the neurobiological underpins of social adaptation by enabling a controlled interference in social organization. More so, it has the complexity inherent to social networks, but it simultaneously allows to longitudinally follow each individual in a number of behavioral parameters. Additional layers of analysis can be associated in future works to better capture the full spectrum of complexity in social interactions.

## Data availability statement

The raw data supporting the conclusions of this article will be made available by the authors, without undue reservation.

## Ethics statement

This animal study was reviewed and approved by ORBEA, Universidade do Minho.

## Author contributions

NSo conceived the experiment and provided the funding. LA, MC-C, SD, LJ, and HL-A performed data collection. SD, LA, and HL-A performed data analysis. SD, LA, LJ, and HL-A wrote a preliminary draft of the manuscript. All authors discussed the data, contributed to the experimental design, and contributed for the final manuscript.
